# The effects of HIV testing advocacy messages on test acceptance: a randomized clinical trial

**DOI:** 10.1186/s12916-014-0204-4

**Published:** 2014-11-06

**Authors:** Monica L Kasting, Anthony D Cox, Dena Cox, Kenneth H Fife, Barry P Katz, Gregory D Zimet

**Affiliations:** Department of Epidemiology, Richard M. Fairbanks School of Public Health, 714 N. Senate Ave. Suite EF250, Indianapolis, IN 46202 USA; Department of Marketing, Indiana University Kelley School of Business, 801 W. Michigan Street, Indianapolis, IN 46202 USA; Department of Medicine, Indiana University School of Medicine, 545 N. Barnhill Dr., Emerson Hall, Indianapolis, IN 46202 USA; Department of Biostatistics, Indiana University School of Medicine and Fairbanks School of Public Health, 410 West 10th Street, Suite 3000, Indianapolis, IN 46202 USA; Department of Pediatrics, Indiana University School of Medicine, 410 West 10th Street, Suite 1001, Indianapolis, IN 46202 USA

**Keywords:** Attitude to health, Health communication, HIV, HIV infections/diagnosis, Intervention studies

## Abstract

**Background:**

Nearly 1 in 5 people living with HIV in the United States are unaware they are infected. Therefore, it is important to develop and evaluate health communication messages that clinicians can use to encourage HIV testing.

**Methods:**

The objective was to evaluate health communication messages designed to increase HIV testing rates among women and evaluate possible moderators of message effect. We used a randomized four-arm clinical trial conducted at urban community outpatient health clinics involving 1,919 female patients, 18 to 64 years old. The four health message intervention groups were: i) information-only control; ii) one-sided message describing the advantages of HIV testing; iii) two-sided message acknowledging a superficial objection to testing (i.e., a 20 minute wait for results) followed by a description of the advantages of testing; and iv) two-sided message acknowledging a serious objection (i.e., fear of testing positive for HIV) followed by a description of the advantages of testing. The main outcome was acceptance of an oral rapid HIV test.

**Results:**

Participants were randomized to receive the control message (n = 483), one-sided message (n = 480), two-sided message with a superficial objection (n = 481), or two-sided message with a serious objection (n = 475). The overall rate of HIV test acceptance was 83%. The two-sided message groups were not significantly different from the controls. The one-sided message group, however, had a lower rate of testing (80%) than the controls (86%) (OR, 0.66; 95% CI, 0.47–0.93; *P* = 0.018). “Perceived obstacles to HIV testing” moderated this effect, indicating that the decrease in HIV test acceptance for the one-sided message group was only statistically significant for those who had reported high levels of obstacles to HIV testing (OR, 0.36; 95% CI, 0.19–0.67; *P* = 0.001).

**Conclusions:**

None of the messages increased test acceptance. The one-sided message decreased acceptance and this effect was particularly true for women with greater perceived obstacles to testing, the very group one would most want to persuade. This finding suggests that efforts to persuade those who are reluctant to get tested, in some circumstances, may have unanticipated negative effects. Other approaches to messaging around HIV testing should be investigated, particularly with diverse, behaviorally high-risk populations.

**Trial registration:**

Clinicaltrials.gov Identifier: NCT00771537. Registration date: October 10. 2008

## Background

The Centers for Disease Control and Prevention (CDC) estimate that there are nearly 1.1 million people living with HIV in the United States today and that nearly 1 in 5 are unaware they are infected [1]. This situation may be explained by a recent report published by the CDC, which indicated that only 45% of adults in the United States have ever been tested for HIV and only 10% to 12% of those had been tested in the last 12 months [2,3]. Higher rates of testing will have to be achieved in order to meet the U.S. Department of Health and Human Services Healthy People 2020 goal of increasing the percentage of HIV-infected persons who know that they are infected from 80.9% to 90% [4]. Early detection of HIV is critical for initiation of treatment to prevent progression to AIDS and for effective viral suppression [5]. Furthermore, people who are aware of their HIV status are more likely to reduce their risky sexual behaviors and are therefore less likely to transmit the virus to their partners [6]. Thus, it is important to evaluate interventions, including health messaging, designed to increase rates of HIV testing.

When recommending a health behavior, one can employ either a one-sided or a two-sided message. A one-sided message presents only arguments supportive of the advocated behavior or position. A two-sided message provides supporting arguments, but also acknowledges (and usually rebuts) one or more potential arguments against the advocated behavior. The instinct of most health professionals is to stress the benefits of the behavior they are advocating; as a consequence, the vast majority of persuasive messages (e.g., advertisements) are one-sided [7]. However, seminal studies by Hovland et al. found that two-sided messages are sometimes more persuasive than one-sided messages [8]. The persuasive superiority of two-sided messages may be more likely to hold if the audience is resistant to persuasion (i.e., holds negative prior attitudes toward the advocated behavior) [7].

The principal theory addressed in this study is Inoculation Theory, which is said to increase the effectiveness of a two-sided message [9]. Inoculation Theory holds that a two-sided message works in much the same way as vaccination; that is, by exposing subjects to a weakened (e.g., rebutted) form of a negative argument. Subjects are believed to be less likely to raise mental “counter-arguments” during message processing than if they had just heard a one-sided message, theoretically leading to increased message acceptance [10-12]. However, it is important to note that Inoculation Theory suggests the effects of a two-sided message may be less effective than a one-sided message if it raises negative arguments that would not otherwise have occurred to the audience. As a result, for some individuals and in some circumstances, a two-sided message may lead to a boomerang effect (i.e., create more negative attitudes than existed prior to message exposure). Furthermore, without the “inoculating” effect of the two-sided message, a one-sided message is likely to generate counter-arguments among resistant audience members (e.g., those who have high perceived barriers to testing), and these counter-arguments may be more persuasive than the message itself, also causing a boomerang effect.

Our objective was to evaluate one-sided and two-sided health communication messages designed to overcome attitudinal barriers with the goal of increasing HIV testing rates among women, who continue to be a significantly underrepresented population in HIV-related research [13]. In addition to evaluating the effects of message sidedness, we were also interested in assessing whether any significant effects of the intervention might vary (i.e., be moderated by) at different levels of socio-demographic factors (age, race/ethnicity, education, income, relationship status, employment status), sexual behavior (total number of sexual partners over the lifetime), and/or HIV testing-related health beliefs.

## Methods

### Procedures and participants

Eligible participants for this study were women who were 18 years of age and older, able to understand English or Spanish, HIV-negative (via self-report), non-pregnant, and seeking clinical services at one of seven urban community health clinics located in Indianapolis, IN, USA. All recruitment sites were full-service health clinics that offered a range of diagnostic and treatment services. Patient demographics varied among the seven different clinic sites with some being fairly equally distributed across Hispanic, non-Hispanic black, and non-Hispanic white while others had higher proportions of either black or white. For the purpose of our study, we oversampled ethnic minorities so that we could assess potential differences in the effects of messaging across different races/ethnicities. Questions, information, and the interventions were presented to the participants one time via audio computer-assisted self-interview (ACASI), so high levels of literacy were not required, and participants only had to be able to recognize individual numbers and letters to indicate their responses. Surveys were completed in a private room and it took each participant an average of 20 minutes to complete the survey. Pregnant women (by self-report) were excluded because HIV testing is routinely recommended and provided to all women who are pregnant.

The study was approved by the Indiana University Institutional Review Board and data were collected from August 2008 to January 2011. Potential participants were recruited from clinic waiting rooms, but all consent and study procedures took place in a private area. All participants provided written informed consent and were compensated with a $25 gift card for the time and effort involved in completing the ACASI. Individuals who met the inclusion criteria and agreed to participate were oriented to the ACASI by the project manager. Throughout the ACASI process the project manager remained available for questions or problems, but did not directly supervise the ACASI.

### Study design

This study followed a four-group parallel design with balanced randomization across four message groups. We randomized adult women into the following groups: i) an information-only control condition; ii) a one-sided message advocating HIV testing; iii) a two-sided message acknowledging and refuting a superficial objection; and iv) a two-sided message acknowledging and refuting a more serious objection. Individuals in the control group received brief, basic information about HIV/AIDS and about the rapid HIV test being offered (this information was provided to all four arms). The one-sided group saw and heard messages describing only the benefits of HIV testing (e.g., “If you detect HIV early, you can get treatments that can greatly improve your long term health”). The two-sided superficial message involved description of a relatively minor objection to testing (e.g., “Some people may not get tested because they think it is inconvenient to wait 20 minutes to get the result”), followed by refutation of the objection and the message noting the benefits of testing. The two-sided serious message involved a more substantial objection to testing (e.g., “Some people do not get tested for HIV because they are afraid that they will find out that they have HIV infection”) followed by refutation of the objection and the message noting the benefits of testing. By preemptively presenting objections to testing, along with refutations of the objections, Inoculation Theory would predict a decrease in resistance to getting tested for HIV. The messages were developed from qualitative interviews conducted during a pilot study for this project. The serious objection was determined from these qualitative interviews carried out at the same clinics used to conduct the surveys [14]. Wait time was not identified as a serious objection by participants in the qualitative study so we determined that it was a good representation of a superficial barrier. Table 1 shows the full text of the four messaging conditions. Using a computer-generated random number algorithm, participants were randomized within the ACASI program in blocks of 64 to one of the four health message conditions. Across each sequential group of 64 participants, 16 would be assigned to each study arm with a 1:1:1:1 allocation ratio. Both research staff and participants were blinded to the intervention.

**Table 1:** **Intervention messages**

**Intervention messages**			
**Control message**	**One-sided message**	**Two-sided message with a superficial objection**	**Two-sided message with a serious objection**
The HIV test tells you if you are infected with HIV, the virus which can cause AIDS. AIDS destroys your body’s ability to fight illness.	The HIV test tells you if you are infected with HIV, the virus which can cause AIDS. AIDS destroys your body’s ability to fight illness.	The HIV test tells you if you are infected with HIV, the virus which can cause AIDS. AIDS destroys your body’s ability to fight illness.	The HIV test tells you if you are infected with HIV, the virus which can cause AIDS. AIDS destroys your body’s ability to fight illness.
	If you get the HIV test, we take a swab of saliva from your mouth, and in about 20 minutes you get the result.	If you get the HIV test, we take a swab of saliva from your mouth, and in about 20 minutes you get the result.	If you get the HIV test, we take a swab of saliva from your mouth, and in about 20 minutes you get the result.
If you get the HIV test, we take a swab of saliva from your mouth, and in about 20 minutes you get the results.	There are many benefits of getting the HIV test:	There are some reasons that people give for not getting the HIV test. For example, some people may not get tested because they think it is inconvenient to wait 20 minutes to get the result.	There are some reasons that people give for not getting the HIV test. Some people do not get tested for HIV because they are afraid that they will find out that they have HIV infection.
	– If you detect HIV early, you can get treatments that can greatly improve your long term health.	But 20 minutes is a fairly short time to wait and there are many benefits of getting the HIV test:	But there are many benefits of getting the HIV test and finding out your results:
	– If you know you have HIV, you can do things to protect the ones you love.	– If you detect HIV early, you can get treatments that can greatly improve your long term health.	– If you detect HIV early, you can get treatments that can greatly improve your long term health.
	– If you are thinking about getting pregnant, you can get treatments to protect the baby from HIV.	– If you know you have HIV, you can do things to protect the ones you love.	– If you know you have HIV, you can do things to protect the ones you love.
	– If the test shows you don’t have HIV, you will feel relieved.	– If you are thinking about getting pregnant, you can get treatments to protect the baby from HIV.	– If you are thinking about getting pregnant, you can get treatments to protect the baby from HIV.
		– If the test shows you don’t have HIV, you will feel relieved.	– If the test shows you don’t have HIV, you will feel relieved.

### Measures and interventions

The ACASI measured socio-demographic factors (age, sex, education, race, employment status, relationship status, and family income). In addition, we assessed potential attitudinal moderators of framing effects with three scales: perceived obstacles to testing, perceived benefits of testing, and normative beliefs about testing. These health beliefs were adapted from previous research on other medical diagnostic tests and vaccine acceptance studies [15-17]. They were assessed with items that used a 5-point Likert-type response scale ranging from ‘Strongly Disagree’ to ‘Strongly Agree’. The perception of obstacles to HIV testing was assessed with a single item (“I can think of a lot of reasons not to get tested for HIV”). Perceived benefits of testing was assessed with five items (e.g., “Getting tested for HIV/AIDS would be a good way to protect my health”) and had good internal reliability (coefficient alpha = 0.81). Normative beliefs about testing were assessed with six items (e.g., “Most of the people I know think that getting tested for HIV/AIDS is a good thing to do for your health”) and had a good internal reliability (alpha coefficient = 0.76).

### Outcome

The outcome of interest was HIV test acceptance. At the conclusion of the survey, participants were offered free oral fluid rapid HIV testing (Oraquick® Test by OraSure Technologies, Inc., Bethlehem, PA, USA). Test acceptance was measured as a binary yes/no variable.

### Statistical analysis

The data were analyzed using logistic regression, with the control message serving as the reference level. First, we analyzed the main effect of the intervention on HIV testing rates. We planned to further analyze any significant main effects with a series of moderation analyses. These analyses were performed in order to determine whether the identified main effect might vary based on subgroups. Potential moderators were age, education, race/ethnicity, employment status, number of lifetime partners, perceived benefits, perceived norms, or perceived obstacles. For the moderation analyses, the designated moderator was added as a predictor, as well as the interaction between the moderator and message type. Any statistically significant moderator analyses (i.e., those that had a statistically significant interaction with message type) were further explored by doing separate logistic regression analyses, stratified by levels of the moderator in order to examine the specific nature of the moderation.

### Power calculations

We enrolled 1,919 participants in the main portion of the study or approximately 480 per experimental condition*.* Testing acceptance in the control group was 86%. Based on a logistic regression model with a two-tailed 5% significance level, we had 88% power to detect a linear effect with a 2% increase with each successive group, i.e., 92% acceptance in the best (expected to be two-sided major objection) message group. For an intervention effect that is not linear, we would still have 93% power to detect a main effect if the groups were evenly spread between 86% and 93.5% acceptance. The main contrasts planned to be examined were comparisons of the control and the intervention groups. We estimated that there was 80% power to detect a 5.7% difference.

## Results

### Sample description

**Figure 1:**
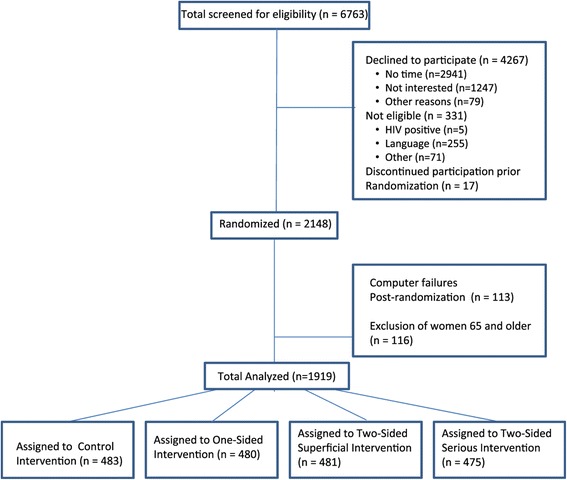
**Flowchart of the study.**

A total of 6,763 women were screened for eligibility and 2,148 were ultimately randomized to the four messaging conditions. Computer and electronic survey failures led to the loss of data from 113 participants (5.3%). In light of the CDC’s recommendation for routine opt-out testing for individuals aged 13 to 64, for this paper we excluded women who were aged 65 years and older (n = 116), resulting in a final sample of 1,919 (Figure 1) [18]^a^. These women were 18 to 64 years of age (mean = 42.7), 20% identified as Latina, 44% identified as non-Latina Black, and 36% identified as non-Latina White/Other. Race was assessed by self-report; Table 2 shows the complete description of socio-demographic characteristics. As shown in Figure 1, participants were distributed fairly equally across intervention conditions and a randomization check indicated that there were no statistically significant differences at baseline across conditions (see columns 2–6 of Table 2). Overall, of the 1,919 participants, 83% accepted the HIV test. Among the women who accepted the HIV test, there was one Western Blot confirmed true positive and one false positive.

**Table 2:** **Sample description by message condition (N = 1,919)**

**Characteristic**	**Total ** **(n = 1919)**	**Control** **(n = 483)**	**One-sided** **(n = 480)**	**Two-sided message with superficial objection ** **(n = 481)**	**Two-sided message with serious objection ** **(n = 475)**	***P*** **value for differences in each category**
Mean age	42.7	42.9	42.9	42.3	42.6	0.868
Education						0.372
<High school	28%	30%	29%	29%	25%	
High school graduate	32%	29%	31%	34%	33%	
>High school education	41%	41%	40%	37%	42%	
Race						0.077
Hispanic	20%	18%	18%	24%	20%	
Non-Hispanic Black	44%	45%	43%	44%	43%	
Non-Hispanic White/Other	36%	37%	39%	31%	37%	
Employment status						0.443
Currently employed	40%	38%	39%	43%	40%	
Currently unemployed	60%	62%	61%	57%	60%	
Relationship status						0.630
Married and living with husband	36%	37%	33%	37%	37%	
Married and not living with husband	10%	12%	9%	11%	8%	
Not married and living with partner	32%	31%	33%	30%	33%	
Not married and not living with partner	23%	20%	26%	22%	22%	
Lifetime sexual partners						0.743
<5	35%	35%	35%	35%	35%	
5–10	36%	37%	35%	37%	33%	
>10	30%	28%	29%	28%	32%	
Annual family income						0.284
<$10,000	47%	47%	47%	48%	45%	
$10,000–$29,999	42%	45%	40%	41%	42%	
≥$30,000	12%	9%	13%	11%	13%	
Perceived obstacles						0.473
Low obstacles	73%	74%	75%	71%	73%	
High obstacles	27%	26%	25%	29%	27%	

### HIV test acceptance

The highest rate of testing (86%) occurred among participants in the control group, who received no persuasive message. Neither the two-sided superficial nor the two-sided serious message group (test acceptance rates 83% and 82%, respectively) differed significantly from the control group (86%) in acceptance of HIV testing. However, the one-sided message group had significantly lower rates of testing (80%) than the control group (86%) (OR, 0.66; 95% CI, 0.47–0.93; *P* = 0.018).

### Moderator analyses

**Figure 2:**
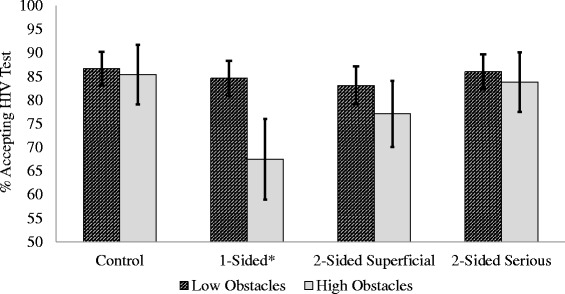
**Moderating effect of perceived obstacles by test acceptance.** *Significant difference in HIV test acceptance between Low and High Perceived Obstacles groups (P = .001).

Of the eight potential moderators, only the interaction between the intervention and “perceived obstacles to testing” was statistically significant (*P* = 0.048). Specifically, compared to the control condition, the one-sided message resulted in lower rates of test acceptance with increases in perceived obstacles (OR, 0.70; 95% CI, 0.53–0.93; *P* = 0.013). To further explain this moderator effect, we dichotomized the sample based on perceived obstacles: low obstacles (i.e., responses of “Strongly Disagree” and “Disagree”) and high obstacles (i.e., responses of “Neither Agree nor Disagree”, “Agree”, and “Strongly Agree”). Logistic regression analyses stratified by level of perceived obstacles indicated no significant effect for participants in the “low perceived obstacles” group, but significantly lower rates of testing with the one-sided message for the “high perceived obstacles” group (OR, 0.36; 95% CI, 0.19–0.67; *P* = 0.001). Table 3 shows the full results of the stratification analysis. Figure 2 shows a graphical representation of the moderating effect of the intervention on test acceptance between those with low and high obstacles.

**Table 3:** **Analysis of messaging effects on HIV testing stratified by level of perceived obstacles**

	**Low obstacles**			**High obstacles**		
	**Percentage accepting HIV test**	**OR (95% CI)**	***P*** **value**	**Percentage accepting HIV test**	**OR (95% CI)**	***P*** **value**
**Intervention:**						
Control	86.7%	n/a		85.4%	n/a	
One-sided	84.6%	0.84 (0.55–1.28)	0.427	67.5%	0.36 (0.19–0.67)*	0001*
Two-sided with superficial objection	83.1%	0.76 (0.50–1.15)	0.187	77.1%	0.58 (0.31–1.09)	0.092
Two-sided with serious objection	86.0%	0.94 (0.61–1.50)	0.782	83.8%	0.89 (0.45–1.76)	0.738

*Statistically significant.

## Discussion

In this study, the women who received either of the two-sided messages did not have HIV testing rates that were significantly higher than those who received information only. Surprisingly, the one-sided message had negative effects on test acceptance, particularly for those women who came into the study perceiving high obstacles to HIV testing. This finding is particularly noteworthy, in that it suggests that efforts at direct persuasion may have the unanticipated effect of discouraging acceptance of HIV testing, particularly among those individuals one would most want to persuade (i.e., those who are reluctant to get tested). Yet, many HIV testing brochures developed by local, state, and federal governments take a largely one-sided approach to health messaging about HIV testing [19-22].

The unanticipated negative effect or “boomerang effect” of the one-sided message that was identified in this study has been described in previous literature on message framing and mammography screening [23,24], suggesting that health messaging interventions need to be carefully evaluated and compared to a control condition. If we had simply tested one-sided vs. two-sided messages, for instance, without an information-only control group, we would not have identified the negative impact of the one-sided condition. Overall, reactance reduced test acceptance by 6%, which is a modest effect size, but meaningful when extrapolated across a large population. However, it is notable that for the high perceived obstacles group, the one-sided message had a very large effect size and reduced test acceptance by nearly 18 percentage points (85.4% vs. 67.5%).

There are a number of limitations to this study which may limit generalizability and suggests that the results should be interpreted with some caution. First, we had a relatively high rate of test acceptance across groups, indicating that a ceiling effect may have limited our ability to increase testing rates with simple health messages. It is possible that two-sided messages might work more effectively in populations with lower base rates of test acceptance. Also, although the study sample was racially/ethnically diverse, it was composed only of women attending clinics in Indianapolis. Moreover, although there was a relatively low participation rate among those screened for eligibility (just over 30%), most of the women who declined did so due to a lack of time, not due to objections about the nature of the study. It is possible that there was a self-selection bias with this study and the participants who were willing to participate in the study were also more willing to get tested for HIV. Furthermore, males, people with differing levels of perceived vulnerability, and those from other geographic locations may respond differently to HIV test messages. A recent study, for instance, found that men and women responded in opposite directions to two-sided messages related to news coverage [25]. In another study, induction of mortality salience increased rates of HIV testing, but only among those who were exposed to a message that increased their feelings of vulnerability to HIV. Mortality salience among participants in the low vulnerability condition resulted in lower rates of testing [26]. It is possible that a brief intervention such as this may work in a population where HIV test acceptance is low. In order to increase test acceptance beyond the already high rate identified in this study may require a more in-depth intervention derived from theories of behavior change such as Social Cognitive Theory [27,28].

## Conclusions

To our knowledge, this is among the first randomized clinical trials examining the effect of persuasive health messages on HIV test acceptance in a clinical setting. We found high rates of test acceptance across health messaging conditions, but no increased testing rates in the intervention groups. In fact, the one-sided persuasive message unexpectedly led to decreased acceptance of testing compared to an information-only control group. This finding suggests that certain approaches to health messaging, commonly used in clinical practice, can actually undermine efforts to maximize rates of HIV testing, particularly among those who express reluctance to get tested. Further research is needed to identify effective health messaging and to evaluate messages in diverse populations.

## Endnote

^a^When women over 65 were included, the results were the same.

## Abbreviations

ACASI: Audio computer-assisted self-interview
CDC: Centers for Disease Control and Prevention
